# Reversal of Cancer Multidrug Resistance (MDR) Mediated by ATP-Binding Cassette Transporter G2 (ABCG2) by AZ-628, a RAF Kinase Inhibitor

**DOI:** 10.3389/fcell.2020.601400

**Published:** 2020-12-08

**Authors:** Jing-Quan Wang, Qiu-Xu Teng, Zi-Ning Lei, Ning Ji, Qingbin Cui, Han Fu, Lizhu Lin, Dong-Hua Yang, Ying-Fang Fan, Zhe-Sheng Chen

**Affiliations:** ^1^Department of Pharmaceutical Sciences, College of Pharmacy and Health Sciences, St. John’s University, Queens, NY, United States; ^2^School of Public Health, Guangzhou Medical University, Guangzhou, China; ^3^Cancer Center, The First Affiliated Hospital of Guangzhou University of Chinese Medicine, Guangzhou, China; ^4^Department of Hepatobiliary Surgery I, Zhujiang Hospital, Southern Medical University, Guangzhou, China

**Keywords:** multidrug resistance, chemotherapy, ABCG2, AZ-628, RAF kinase inhibitor

## Abstract

Overexpression of ABCG2 remains a major impediment to successful cancer treatment, because ABCG2 functions as an efflux pump of chemotherapeutic agents and causes clinical multidrug resistance (MDR). Therefore, it is important to uncover effective modulators to circumvent ABCG2-mediated MDR in cancers. In this study, we reported that AZ-628, a RAF kinase inhibitor, effectively antagonizes ABCG2-mediated MDR *in vitro*. Our results showed that AZ-628 completely reversed ABCG2-mediated MDR at a non-toxic concentration (3 μM) without affecting ABCB1-, ABCC1-, or ABCC10 mediated MDR. Further studies revealed that the reversal mechanism was by attenuating ABCG2-mediated efflux and increasing intracellular accumulation of ABCG2 substrate drugs. Moreover, AZ-628 stimulated ABCG2-associated ATPase activity in a concentration-dependent manner. Docking and molecular dynamics simulation analysis showed that AZ-628 binds to the same site as ABCG2 substrate drugs with higher score. Taken together, our studies indicate that AZ-628 could be used in combination chemotherapy against ABCG2-mediated MDR in cancers.

## Introduction

Multidrug resistance (MDR) is a major barrier of successful chemotherapy (Gottesman and Ambudkar, 2001). A number of mechanisms are associated with MDR in cancer cells including DNA repair and damage, altered drug metabolism, inhibition of apoptosis and overexpression of efflux transporters (Zhang et al., 2015; Lage, 2016; Cui et al., 2019b). Among the potential mechanisms, up-regulation of ATP-binding cassette (ABC) transporters, energized by ATP hydrolysis to mediate active transportation of substrate drugs, is the major one in MDR cancer cells (Borst and Elferink, 2002; Teodori et al., 2006; Cui et al., 2018).

ATP-binding cassette transporters, located on cell membrane, belong to a superfamily which composes of 49 members and is classified into 7 subfamilies from A to G (Dlugosz and Janecka, 2016). Among them, ABCB1, ABCG2, and ABCCs have been widely reported as MDR inducers in cancer cells (Morrow, 2006; Ween et al., 2015; Taylor et al., 2017). As an important member of ABC transporters, ABCG2 (breast cancer resistance protein or BCRP) has been well studied since its first discovery (Robey et al., 2001). ABCG2 expresses ubiquitously in organs and tissues including small intestine, liver, colon, and placenta, where ABCG2 plays the role of protecting normal cells from accumulation of toxins (Honjo et al., 2001; Zaher et al., 2006; Mao and Unadkat, 2015). However, the efflux function of ABCG2 causes deleterious effects in cancer cells: it decreases intracellular accumulation of anticancer drugs which leads to lower chemotherapeutic efficacy (Honjo et al., 2001; Polgar et al., 2008, 2; Taylor et al., 2017, 2). Clinically, ABCG2 has been detected in various cancer cells including breast, lung, bladder, and colon cancers (Mao and Unadkat, 2015). High ABCG2 expression was also reported in late stages (stage III and state IV) cancers that showed MDR (Friedrich et al., 2004). Common substrates of ABCG2 include mitoxantrone, SN-38, and topotecan (Zaher et al., 2006).

Recently, various small molecule drugs as well as peptides were found to be potent modulators/substrates of ABCB1-, ABCG2-, or ABCCs-mediated MDR (Cui et al., 2019a; Liao et al., 2019; Wang et al., 2019, 2020b; Wu et al., 2019, 2020c; Luo et al., 2020; Teng et al., 2020). The overexpression of ABCG2 has been found to be related to acquired MDR in multiple types of cancers including breast cancer (Mao and Unadkat, 2015), non-small cell lung cancer (Yoh et al., 2004) and acute myelogenous leukemia (Ross et al., 2000). Instead of designing and synthesizing novel ABCG2 inhibitors, we have focused on the repurposing small-molecule targeted therapeutic agents as ABCG2 modulators as an alternative approach to overcome MDR (Wu et al., 2020a; Zhang et al., 2020b). As a potent and selective RAF kinase inhibitor, AZ-628 has showed promising preclinical results (Halilovic and Solit, 2008). Moreover, AZ-628 showed exquisite efficacy in cell lines harboring the V600E BRAF mutations compared with sorafenib, a FDA-approved RAF kinase inhibitor for renal and hepatocellular carcinoma (McDermott et al., 2007). In this study, we reported that AZ-628 significantly sensitized ABCG2-mediated MDR to ABCG2 substrate drugs at a non-toxic concentration in cancer cells.

A recent study revealed that overexpression of ABC transporters was responsible for acquired resistance to antibody-drug conjugate (ADC) in cancer immunotherapy, and combinational administration of ABC transporter inhibitors increased the response rate of immunotherapy in resistant cancer cells (Chen et al., 2020). Moreover, such combinational therapy has been subjected to a phase I clinical trial (Chen et al., 2020). Therefore, finding novel ABC transporter inhibitors is critical for preclinical and clinical cancer research.

## Materials and Methods

### Chemicals

AZ-628 was purchased from Selleckchem (Houston, TX, United States). Chemotherapeutic agents including mitoxantrone, topotecan, SN-38, paclitaxel, and cisplatin were purchased from Sigma Co. (St. Louis, MO, United States). Cell culture reagents including bovine serum, fetal bovine serum, DMEM and 0.25% trypsin were purchased from Corning Inc. (Tewksbury, MA, United States). Immunoblotting materials including human ABCG2 monoclonal antibodies for ABCG2 and GAPDH, Alexa Fluor 488 conjugated antibody and HRP-conjugated secondary antibody were purchased from Millipore (Billerica, MA, United States). Tritium-labeled mitoxantrone ([^3^H]-mitoxantrone, 2.5 Ci/mmol) was purchased from Moravek Biochemicals, Inc. (Brea, CA, United States).

### Cell Lines

Non-small cell lung cancer cell line H460 and mitoxantrone-selected MDR cell line H460/MX20, human colon adenocarcinoma cell line S1 and mitoxantrone-selected MDR cell line S1-M1-80 and human epidermoid carcinoma cell line KB-3-1 and colchicine-selected MDR cell line KB-C2 and vincristine-selected KB-CV60 were used in this study. Additionally, human embryonic kidney cell line HEK293 transfected with empty vector pcDNA3.1 or pcDNA3.1-ABCG2 (wild-type or mutants) was used. H460/MX20 cells were maintained using 20 μM mitoxantrone. KB-C2 cells were maintained using 2 μg/ml colchicine. KB-CV60 cells were maintained using 1 μg/ml cepharanthine and 60 ng/ml vincristine. All HEK293 transfected cells were screened using 2 mg/ml G418. All cell lines were cultured in DMEM with 10% FBS and 1% P/S in a humidified environment with 5% CO2 at 37°C. All drug-selected resistant cells were cultured in drug-free medium at least 2 weeks before experiment.

### Cytotoxicity Assay

Cytotoxicity of anticancer drugs was measured by modified MTT-based colorimetric assay as previously described (Wang et al., 2020b). For reversal study, the reversing effect was measured by altered IC50. Briefly, cells at the density of around 5,000 cells/well were seeded equally into 96-well plates. After overnight culture, 1 or 3 μM AZ-628 or a positive ABCG2 modulator (3 μM KO143) were added and incubated for 2 h. Subsequently, gradient concentrations of anticancer drugs were added and incubated for 68 h, followed by adding 20 μl 4 mg/ml MTT in each well and incubating for an additional 4 h. Then 100 μl DMSO was added in each well and plates were shaken until purple formazan was completely dissolved. Absorbance at 570 nm was measured and IC50 values were calculated as previously described (Zhang et al., 2020b).

### Western Blotting

Western blotting was performed to determine protein expression level. Western blotting was performed as previously described (Wu et al., 2020b)s with minor modification. Cells were divided into two groups for concentration- or time-dependent study. In concentration-dependent study, different concentrations of AZ-628 (0, 1, and 3 μM) were added and incubated with H460 or H460/MX20 cells for 72 h. In time-dependent study, H460 or H460/MX20 was incubated with 3 μM AZ-628 for 0, 24, 48, or 72 h. Cells were lysed and lysates were collected at the end of each time point. Protein concentration was determined using BCA protein assay and protein was then loaded equally into a 10% sodium dodecyl sulfate-polyacrylamide gel and transferred to a PVDF membrane. Expression of ABCG2 were determined by antibody BXP-21 (1:1000). GAPDH was used as a loading control. Relative expression was quantified using Image J software.

### Immunofluorescence Assay

Immunofluorescence assay was performed to determine protein intracellular localization. Immunofluorescence was performed as previously described (Cai et al., 2020). Briefly, cells were seeded equally to 24-well plates and cultured overnight before adding drugs. Similarly, cells were divided into concentration- and time-dependent study groups and add AZ-628 accordingly. After culturing for 0, 24, 48, or 72 h, cells were subjected to fixation by 4% paraformaldehyde and permeabilization by 0.25% Triton X-100. Then cells were blocked with 6% BSA before incubated with monoclonal human ABCG2 antibody BXP-21 (1:200). Then cells were incubated with secondary antibody conjugated with Fluor 488 (1:1000). Nucluei was counterstained by DAPI (1:1000). Images were taken with an EVOS FL fluorescence microscope (Life Technologies Corporation, MD, United States).

### Intracellular Drug Accumulation Assay

Intracellular accumulation of [^3^H]-mitoxantrone was determined as previously described (Zhang et al., 2018). Briefly, cells were seeded in 24-well plates (100,000 cells/well) 1 day before experiment. Then ABCG2 modulators (AZ-628 at 1 or 3 μM; or KO143 at 3 μM) was added 2 h prior to adding [^3^H]-mitoxantrone. Subsequently, cells were incubated with or without ABCG2 modulators for additional 2 h to get final intracellular concentration of [^3^H]-mitoxantrone. Cells were collected and transferred into scintillation liquid, then radioactivity was measured in the Packard TRI-CARB 1900CA liquid scintillation analyzer (Packard Instrument, Downers Grove, IL, United States).

### Drug Efflux Assay

[^3^H]-mitoxantrone efflux assay was performed as previously described (Ji et al., 2019b). In brief, cells were seeded equally in 24-well plates at a final density of 100,000 cells/well. After culturing overnight, ABCG2 modulators (AZ-628 at 1 or 3 μM; or KO143 at 3 μM) were added 2 h before adding radioactive mitoxantrone. After additional incubation for 2 h, medium was aspirated and radioactive-free medium was added. At each time point (0, 30, 90, and 120 min), cells were collected and transferred into scintillation liquid. Radioactivity was measured as mentioned in the previous section.

### ATPase Assay

The ABCG2-associated ATPase activity was measured as previously described (Ji et al., 2018). Membranes were prepared using protein extraction kit (Qproteome Plasma Membrane Protein Kit, Qiagen) from transfected ABCG2 overexpression HEK293 cells. The membranes were incubated with ATPase buffer (Ji et al., 2019a) at 37°C for 3 min with or without 0.4 mM sodium vanadate. Gradient concentrations of AZ-628, topotecan or M3814 were added and incubated at 37°C for 5 min then 25 mM Mg-ATP solution were added and incubated at 37°C for 20 min. Reactions were terminated by adding 5% SDS. The amount of inorganic phosphate was quantified using a colorimetric method under 880 nm.

### Docking Simulation

ATP-binding cassette transporter G2 protein model was downloaded from RCSB PDB (6ETI, wild-type, resolution 3.1 Å) (Jackson et al., 2018). The 3D structure of AZ-628 was downloaded from PubChem and prepared as previously described (Cai et al., 2019). Docking calculations were performed in AutoDock Vina (1.1.2) (Trott and Olson, 2009). The protein model and the model of AZ-628 were modified by adding hydrogen atoms and partial charges in AutoDockTools (1.5.4). The results of docking simulation and ligand-receptor interactions were visualized in PyMOL (2.3, non-commercial version). Grid center and size were determined according to the original ligands in 6ETI. All docking simulations were performed using default settings. The conformation of AZ-628 with highest affinity score was exported for visualization and further analysis. Docking computations were performed in a 4-core CPU with mac Mojave system.

### Molecular Dynamics (MD) Simulation

The top-scored AZ-628 docked into ABCG2 model was used for MD simulation as previously described (Yang et al., 2020b). In brief, AZ-628-ABCG2 complex model was loaded into Desmond developed by DE Shaw Research Group. The protein-membrane-solvent system was established using Maestro Module System Builder with POPC bilayer membrane, TIP3P water and ions for neutralization. Afterward, the system was subjected to a 50 ns MD simulation with constant temperature (300 K) and pressure (1.015 bar pressure). MD simulation results were analyzed and visualized in an Ubuntu 18.04 system with an NVIDIA^®^ Tesla P100 GPU.

### Statistics

All data in this study was generated from at least three independent triplicated experiments. All results were presented as mean ± SD. Significance were calculated using one-way ANOVA followed by *post hoc* analysis.

## Results

### Cytotoxicity of AZ-628 in Drug Selected and Transfected MDR Cell Lines

**FIGURE 1:**
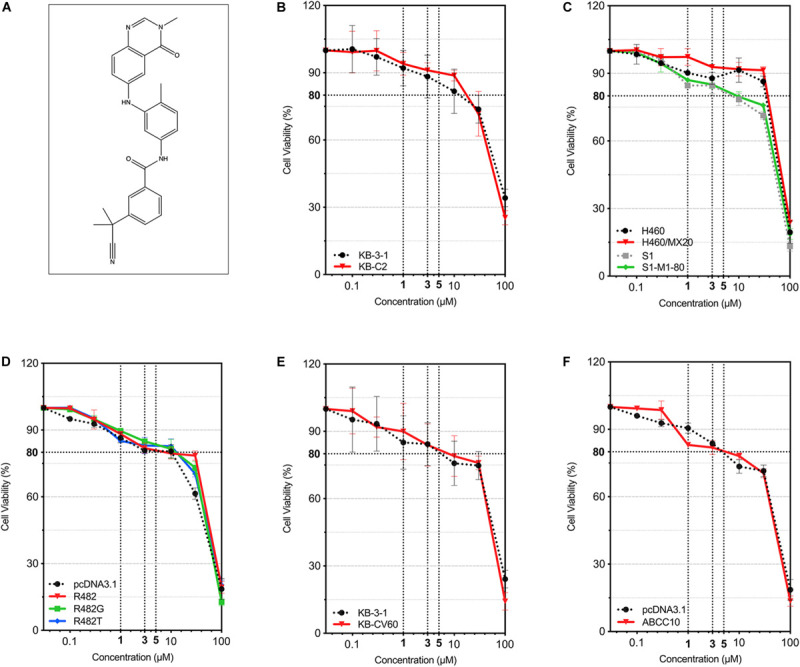
Chemical structure of AZ-628 and its cytotoxicity in different cell lines. **(A)** 2-D Chemical structure of AZ-628. **(B–F)** Cytotoxicity of AZ-628 in parental and MDR cells. Cell viabilities (survival rates) at different concentrations of AZ-628 (0–100 μM) were plotted. 80% cell viability as well as 1, 3, and 5 μM were showed as dashed lines. Points with error bar represent mean ± SD.

The cytotoxicity of AZ-628 on different parental and MDR cell lines was tested. For ABCB1-mediated MDR cancer cells, we used KB-3-1 (parental) and KB-C2 (colchicine-selected ABCB1-mediated MDR cells). For ABCG2-mediated MDR cells, we used S1, H460 (parental) and S1-M1-80, H460/MX20 (mitoxantrone-selected ABCG2-mediated MDR cells), as well as HEK293 transfected with empty vector (HEK293/pcDNA3.1) or recombinant vector containing full-length wild-type/mutant human ABCG2 gene (HEK293/ABCG2-R482, HEK293/ABCG2-R482G, or HEK293/ABCG2-R482T). For ABCC1-mediated MDR cancer cells, we used KB-3-1 (parental) and KB-CV60 (cepharanthine + vincristine-selected ABCC1-mediated MDR cells). For ABCC10-mediated MDR cells, we used HEK293 transfected with empty vector (HEK293/pcDNA3.1) or recombinant vector containing full-length human ABCC10 gene (HEK293/ABCC10). Chemical structure of AZ-628 was given in Figure 1A. According to the results in Figures 1B–F, AZ-628 showed low cytotoxicity in all cell lines with the IC_50_ over 50 μM. Moreover, the IC_50_ between parental and resistant cells showed no significant difference, which indicated that AZ-628 is not a substrate of ABCB1, ABCG2, ABCC1, or ABCC10. Also, we chose the non-toxic concentrations (1 and 3 μM) of AZ-628 by its IC_20_.

### Sensitization of ABCG2-Mediated MDR to Anticancer Drugs by AZ-628

After determining the non-toxic concentration of AZ-628, we then studied whether AZ-628 can affect the MDR in cancer cells which overexpress wild-type or mutant ABCG2 proteins. Based on the results displayed in Figure 2, AZ-628 at the concentration of 1 or 3 μM significantly reversed the resistance to mitoxantrone, SN-38 and topotecan in H460/MX20 (Figures 2A,C,E) and S1-M1-80 (Figures 2B,D,F) cells. It is noteworthy that AZ-628 at 3 μM showed better reversal effects than the positive ABCG2 modulator KO143 in both H460/MX20 and S1-M1-80. AZ-628 did not alter the cytotoxicity of cisplatin, a non-substrate of ABCG2.

**FIGURE 2:**
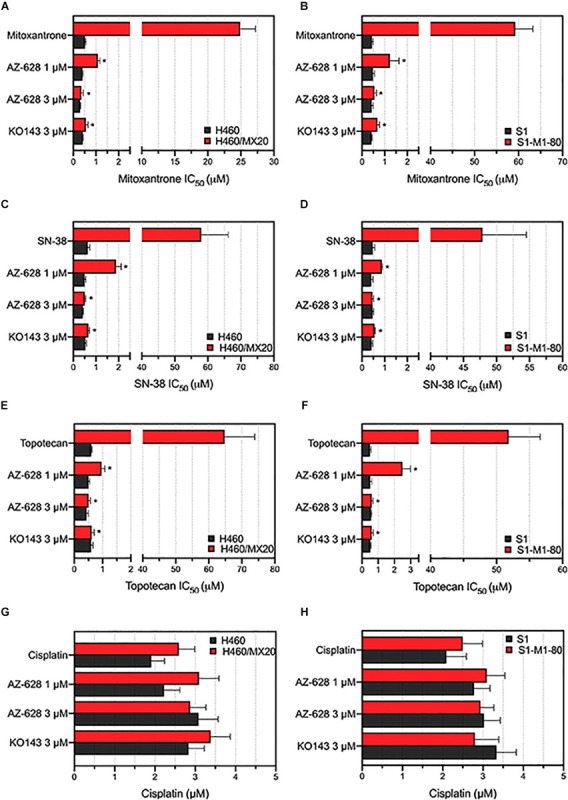
Effects of AZ-628 in ABCG2-overexpressing drug-selected cancer cells. Sensitization of mitoxantrone resistance by AZ-628 in **(A)** H460/MX20 and **(B)** S1-M1-80 cells. Sensitization of SN-38 resistance by AZ-628 in **(C)** H460/MX20 and **(D)** S1-M1-80 cells. Sensitization of topotecan resistance by AZ-628 in **(E)** H460/MX20 and **(F)** S1-M1-80 cells. Effects of AZ-628 on **(G)** H460/MX20 and **(H)** S1-M1-80 cells when incubated with cisplatin. Parental cells (H460 or S1) were used as drug sensitive control cell lines. Columns and error bars represent mean ± SD. Statistical significance (*) was determined by *p* < 0.05.

**FIGURE 3:**
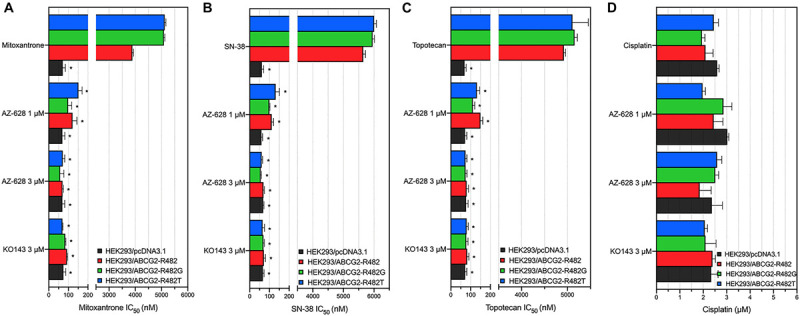
Effects of AZ-628 in ABCG2-overexpressing transfected cells. Sensitization of **(A)** mitoxantrone **(B)** SN-38 or **(C)** topotecan resistance by AZ-628 (1 or 3 μM) in HEK293/ABCG2-R482 (wild-type), HEK293/ABCG2-R482G (mutant), and HEK293/ABCG2-R482T (mutant). Parental cell line (HEK293/pcDNA3.1) was used as sensitive control cell line. **(D)** Cisplatin, a non-substrate of ABCG2, as a negative control anticancer drug. Columns and error bars represent mean ± SD. Statistical significance (*) was determined by *p* < 0.05.

To further determine that the sensitization caused by adding AZ-628 was related to ABCG2, we chose transfected HEK293 cells to verify the reversal effects of AZ-628 since ABCG2 would be considered a single factor contributing to MDR to ABCG2-substrate drugs. Furthermore, we tested the effects of AZ-628 in transfected ABCG2-mediated MDR cells. According to the results in Figure 3, AZ-628 showed significant sensitization effect of different ABCG2 substrates in both wild-type and mutant ABCG2-overexpressing transfected cells. Again, AZ-628 did not alter the cytotoxicity of cisplatin in transfected cells. Similarly, AZ-628 at 3 μM showed comparative reversal effects as KO143 at same concentration.

**FIGURE 4:**
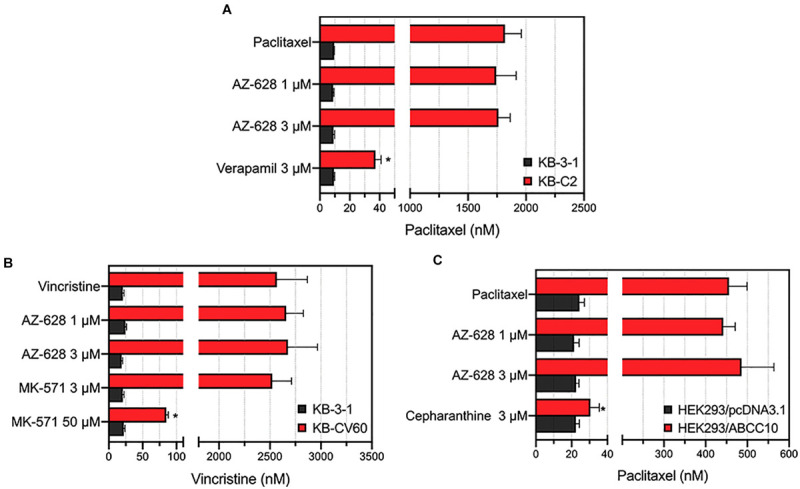
Effects of AZ-628 in ABCB1-, ABCC1-, and ABCC10-overexpressing cells. **(A)** Effects of AZ-628 on KB-3-1 (parental) and KB-C2 (ABCB1-overexpression) cells. Verapamil was used as a positive ABCB1 inhibitor. **(B)** Effects of AZ-628 on KB-3-1 (parental) and KB-CV60 (ABCC1-overexpression) cells. MK-571 was used as a positive ABCC1 inhibitor. **(C)** Effects of AZ-628 on HEK293/pcDNA3.1 (parental) and HEK293/ABCC10 (ABCC10-overexpression) cells. Cepharanthine was used as a positive ABCC10 inhibitor. Columns and error bars represent mean ± SD. Statistical significance (*) was determined by *p* < 0.05.

To further explore the reversal effects of AZ-628, we tested its effect on ABCB1-, ABCC1-, and ABCC10-overexpressing MDR cells. The results showed that AZ-628 at 1 or 3 μM did not significantly alter the MDR mediated by ABCB1 (Figure 4A), ABCC1 (Figure 4B), or ABCC10 (Figure 4C). These results suggest that the reversal effect of AZ-628 against ABCG2 is selective and comparable to the positive ABCG2 modulator KO143.

### Effects of AZ-628 on ABCG2 Protein Expression and Intracellular Localization

**FIGURE 5:**
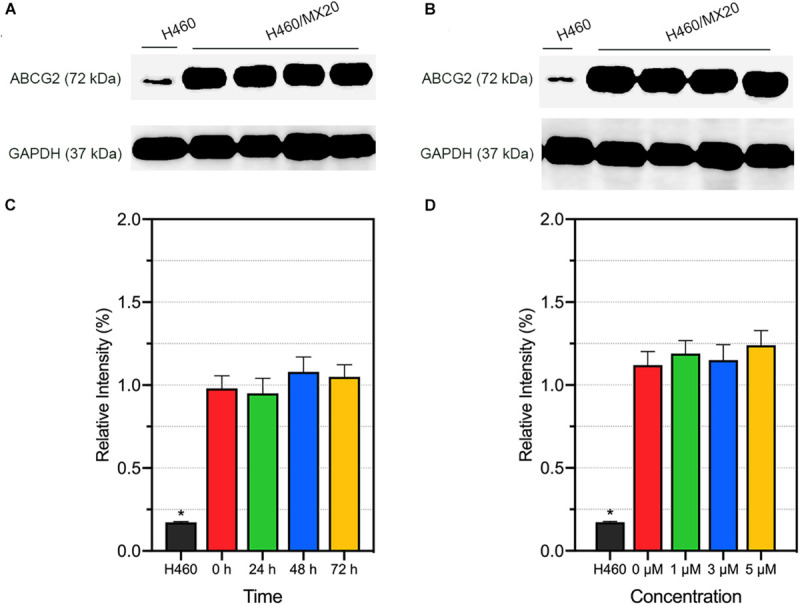
Effects of AZ-628 on ABCG2 protein expression in H460/MX20 cells. ABCG2 and GAPDH (loading control) was marked in **(A,B)**. Ratio of gray intensity was quantified and displayed in **(C,D)**. **(A)** AZ-628 (3 μM) was incubated with different time point (0, 24, 48, or 72 h). **(B)** AZ-628 was incubated with different concentrations (0, 1, 3, or 5 mM) for 72 h. Columns and error bars represent mean ± SD. Statistical significance (*) was determined by *p* < 0.05.

Alteration of ABCG2 protein expression level is one of the possible mechanism of modulating ABCG2-mediated MDR. We performed Western blotting to quantify the ABCG2 protein expression level after incubation with different concentrations (or different incubation time) of AZ-628. It was found that AZ-628 did not significantly alter ABCG2 expression level after incubating cells with different time (0, 24, 48, and 72 h) or concentrations (0, 1, 3, and 5 μM) of AZ-628 (Figure 5). This result suggests that the reversal mechanism of AZ-628 is not related to altering ABCG2 protein expression level.

**FIGURE 6:**
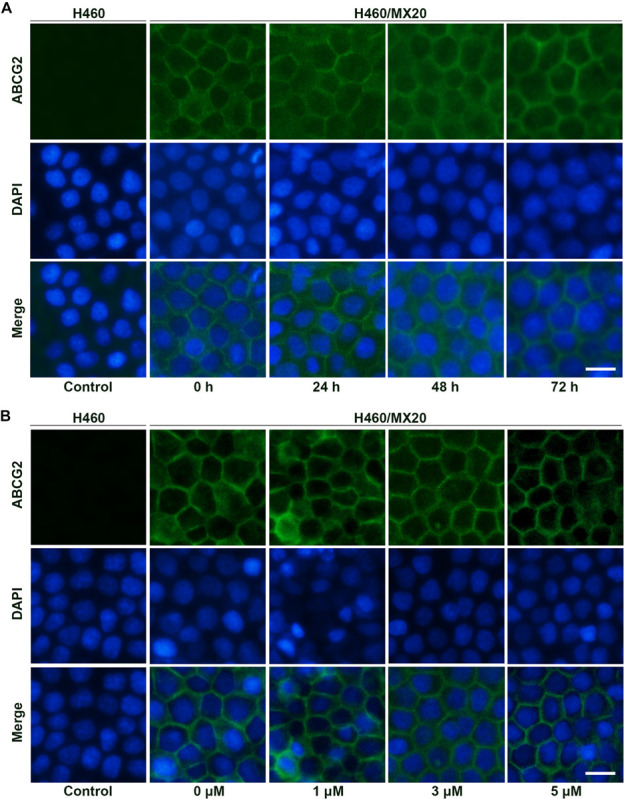
Effects of AZ-628 on ABCG2 intracellular localization. **(A)** Intracellular localization of ABCG2 in H460 or H460/MX20 after incubation with 3 μM AZ-628 for 0, 24, 48, or 72 h. **(B)** Intracellular localization of ABCG2 in H460 or H460/MX20 after incubation with 0, 1, 3, or 5 μM AZ-628 for 72 h. Scale bar indicates 200 μm. Green, ABCG2; Blue, nuclei stained by DAPI.

Besides protein expression, the reversal effects could also be caused by alteration of intracellular localization. As a membrane protein, ABCG2 will lose biological functions if detached from plasma membrane. To determine the intracellular localization of ABCG2 protein after incubation with different time (3 μM; 0, 24, 48, and 72 h) or different concentrations of AZ-628 (72 h; 0, 1, 3, and 5 μM), we performed immunofluorescence staining. According to the results in Figure 6, AZ-628, with different concentrations or incubation time, did not significantly alter the intracellular localization of ABCG2 protein in H460/MX20 cells.

### Effects of AZ-628 on the Intracellular Accumulation of Mitoxantrone

**FIGURE 7:**
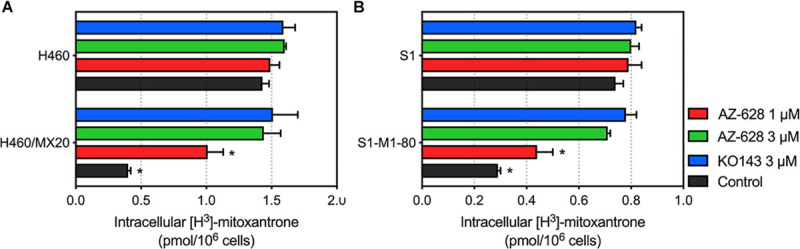
**(A)** Effects of AZ-628 on intracellular accumulation of [^3^H]-mitoxantrone in H460 and H460/MX20. **(B)** Effects of AZ-628 (1 or 3 mM) on intracellular accumulation of [^3^H]-mitoxantrone in S1 and S1-M1-80. KO 143 was used as a positive control inhibitor of ABCG2. Columns and error bars represent mean ± SD. Statistical significance (*) was determined by *p* < 0.05.

To further understand the mechanism of the reversal effect of AZ-628 on ABCG2-mediated MDR, we conducted an intracellular accumulation assay to evaluate the impact of AZ-628 on accumulation of anticancer drug. As shown in Figure 7, incubation with 3 μM of AZ-628 significantly increased the intracellular accumulation of ABCG2-substrate [^3^H]-mitoxantrone in both ABCG2-overexpressing cancer cells H460/MX20 and S1-M1-80. The results demonstrate that AZ-628 increases the intracellular accumulation of ABCG2-substrate anticancer drug, which explains the reversal effects of AZ-628 on ABCG2-mediated MDR.

### Effects of AZ-628 on the Efflux Function of ABCG2 Protein

**FIGURE 8:**
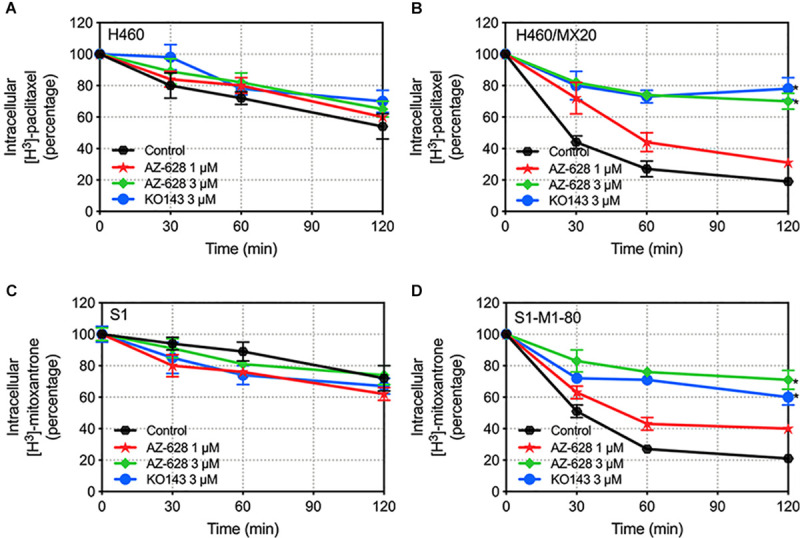
Effects of AZ-628 on the efflux of [^3^H]-mitoxantrone in drug-selected ABCG2-overexpressing cells. Time versus intracellular [^3^H]-mitoxantrone percentage was plotted to reflect the efflux of [^3^H]-mitoxantrone by ABCG2 in **(A)** H460 **(B)** H460/MX20 **(C)** S1 and **(D)** S1-M1-80 cells. KO 143 was used as a positive control inhibitor of ABCG2. Points with error bars represent mean ± SD. Statistical significance (*) was determined by *p* < 0.05.

The above study indicates that AZ-628 increases intracellular accumulation of ABCG2-substrate anticancer drug. To further explore the relationship between increased accumulation and blockage of ABCG2 function, we evaluated the efflux of [^3^H]-mitoxantrone in parental and ABCG2-overexpressing cells. We found that 3 μM of AZ-628 significantly inhibited the efflux function of ABCG2 in H460/MX20 (Figure 8B) and S1-M1-80 (Figure 8D) compared to control groups (Figures 8A,C). These results suggest that increased intracellular accumulation of [^3^H]-mitoxantrone is due to the blockage of ABCG2 efflux function by AZ-628.

### Effects of AZ-628 on ABCG2-Associated ATPase Activity

**FIGURE 9:**
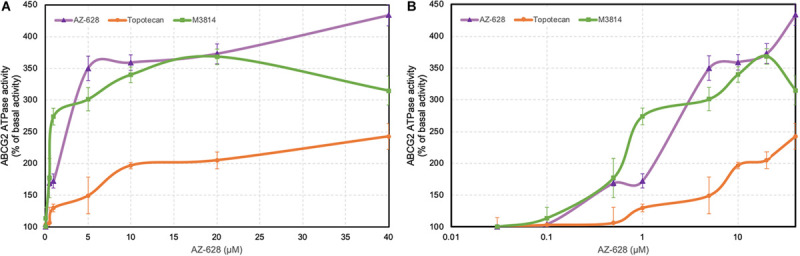
Effects of AZ-628 on ABCG2-associated ATPase activity. **(A)** ABCG2 ATPase activity with different concentrations of AZ-628 (purple), M3814 (green), and topotecan (orange) (0–40 μM). **(B)** ABCG2 ATPase activity with log concentrations of AZ-628, M3814 and topotecan (0–40 μM). Points with error bars represent mean ± SD.

The transportation function of ABCG2 relies on the energy from ATP catalyzed by ABCG2-associated ATPase. Thus altered ABCG2 function by reversal agents could be relevant to ATPase activity (Gottesman and Ambudkar, 2001; Cui et al., 2018). ATPase activity was measured and the results showed that AZ-628 stimulated the ABCG2 ATPase activity (Figure 9), indicating that AZ-628 may compete with substrate anticancer drugs in the binding pockets of ABCG2. Compared with the ABCG2 substrate topotecan, which is also an ABCG2 ATPase stimulator, AZ-628 showed stronger activation effects on ABCG2 ATPase. We also performed ATPase using a recently discovered ABCG2 inhibitor M3814, a potent DNA-PK inhibitor which could reverse ABCG2-mediated MDR efficiently (Wu et al., 2020b). The results showed that AZ-628 stimulated ABCG2 ATPase to a higher level than M3814.

### Interactions Between AZ-628 and ABCG2 by Docking and Molecular Dynamics (MD) Simulation

**FIGURE 10:**
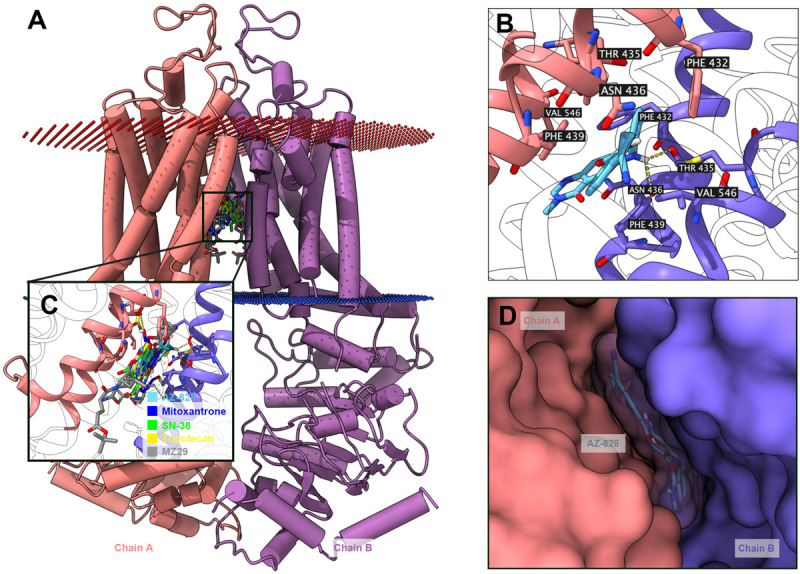
Docking simulation of AZ-628 with human ABCG2 protein model. **(A)** An overview of docking simulation results. ABCG2 was displayed as colored 3-D tube model. Chain A was colored orange and chain B was colored purple. Bilayer cytoplasm membrane was showed as dotted planes. Red plane indicates outer layer and blue plane indicates inner layer. Top-scored ABCG2 substrate drugs and AZ-628 was displayed as colored sticks (blue: mitoxantrone; green: SN-38; yellow: topotecan; sky blue: AZ-628). The co-crystalized ABCG2 inhibitor ZM29 was depicted as gray sticks. **(B)** Detailed interactions of AZ-628 with ABCG2 binding pockets. AZ-628 molecule was colored by heteroatoms. Important amino acids surrounding AZ-628 was labeled and colored by heteroatoms. Hydrogen bond was displayed as yellow dash line. Heteroatoms: red-oxygen, blue-nitrogen, white-hydrogen, carbon-sky blue (AZ-628) or yellow (ABCG2). **(C)** Docked positions of AZ-628 and ABCG2-substrate or ABCG2-inhibitor drugs. Color codes are the same as **(A)**. **(D)** AZ-628 docked in ABCG2 binding pocket with molecule surface displayed.

The above results shown that AZ-628 significantly reversed ABCG2-mediated MDR by increasing intracellular accumulation of chemotherapeutic drugs by blocking the efflux function of ABCG2. Moreover, we found that AZ-628 stimulated ABCG2-associated ATPase activity which indicates that AZ-628 may compete with substrates in ABCG2 binding pocket. However, the accurate interaction between AZ-628 and drug binding site is still unclear. We conducted molecular docking simulation to analyze the potential inter-molecular interactions. The results suggested that AZ-628 could interact with the binding site of ABCG2 with a score of −12.4 kcal/mol. Hydrogen bonds were predicted between the amide group of AZ-628 and THR435 of ABCG2. Besides, AZ-628 was stabilized by hydrophobic pockets formed by PHE439, ASN436, PHE432, MET549, and VAL546 (Figures 10B,D). We also performed docking simulation of ABCG2 substrate drugs mitoxantrone, SN-38 and topotecan. As shown in Figures 10A,C, AZ-628 occupies the same binding site with higher docking score compare with mitoxantrone (−10.2 kcal/mol), SN-38 (−11.0 kcal/mol) as well as topotecan (−10.8 kcal/mol). These results further demonstrate that AZ-628 competes with ABCG2-substrates and affect ABCG2 function. Additionally, 6ETI provided a co-crystalized ABCG2 inhibitor ZM29 in the protein structure (Jackson et al., 2018), which was also visualized in Figures 10A,C. The poses indicated significant overlapping between the binding position of AZ-628 and ZM29.

**FIGURE 11:**
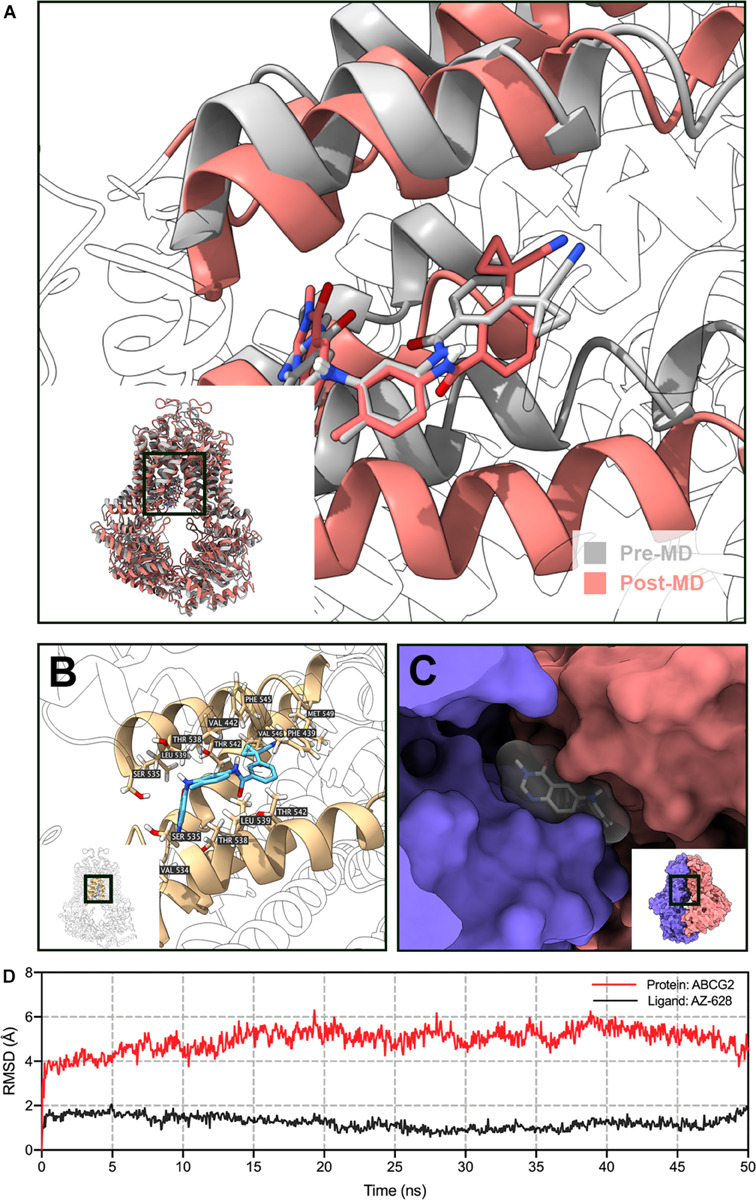
MD simulation (50 ns) of AZ-628 and human ABCG2 model. **(A)** Pre- and post-MD poses of ABCG2 was displayed as gray and red ribbons, respectively. Pre- and post-MD poses of AZ-628 was displayed as gray and red sticks, respectively. For ABCG2, only surrounding ribbons were colored. **(B)** Post-MD pose of AZ-628-ABCG2 complex. AZ-628 was displayed as purple sticks. Important amino acids were labeled and displayed as yellow sticks. For ABCG2, only surrounding ribbons were colored. **(C)** Post-MD pose of AZ-628 and ABCG2 with molecular surface displayed. Post-MD pose of AZ-628 was color gray. **(D)** Root mean square deviation (RMSD) of ABCG2 (red) and AZ-628 (purple) versus reference time (ns).

To further validate the docked poses, a 50 ns MD simulation was performed to evaluate the stability of binding. As shown in Figure 11D, the protein backbone deviated to around 4 Å in the first 2 ns, then it reached a relatively stable conformation till the end of the simulation. Similarly, the fluctuation of AZ-628 was stabilized at the first 2 ns and stabilized at a deviation of 2 Å. Post-MD AZ-628-ABCG2 complex showed that the binding pocket of ABCG2 appeared till the end of simulation. Also, AZ-628 stayed in the same binding pocket through the simulation process (Figures 11A,C). The post-MD complex also showed that AZ-628 was stabilized by similar residues (SER535, THR538, LEU539, THR542, PHE545, VAL546, and MET549) as pre-MD complex (Figure 11B). These results indicate stable interaction between AZ-628 and ABCG2 binding pocket.

## Discussion

Currently, the development of MDR during cancer chemotherapy associated with up-regulation of ABC transporters remains a major issue in cancer treatment (Ullah, 2008; Kovalev et al., 2013). The overexpression of ABCG2 has been found to confer MDR in multiple types of cancers including breast cancer (Mao and Unadkat, 2015), non-small cell lung cancer (Yoh et al., 2004) and acute myelogenous leukemia (Ross et al., 2000). Recently, development of novel ABC transporter inhibitors has achieved promising results in multiple *in vitro* and *in vivo* studies, either via synthesizing new small-molecule compounds (Narayanan et al., 2020; Wang et al., 2020c) or testing the potential reversal effects of FDA-approved drugs (Dong et al., 2020; Feng et al., 2020; Yang et al., 2020a; Wu et al., 2020a). Instead of designing and synthesizing novel ABCG2 inhibitors, we have focused on the repurposing small-molecule targeted therapeutic agents as ABCG2 modulators as an alternative approach to overcome MDR. As a potent and selective RAF kinase inhibitor, AZ-628 has showed promising preclinical results (Halilovic and Solit, 2008). Moreover, AZ-628 showed exquisite efficacy in cell lines harboring the V600E BRAF mutations compared with sorafenib, a FDA-approved RAF kinase inhibitor for renal and hepatocellular carcinoma (McDermott et al., 2007). In this study, we reported that AZ-628 significantly sensitized ABCG2-mediated MDR to ABCG2 substrate drugs at a non-toxic concentration in cancer cells.

As evident from the MTT results, AZ-628 at 3 μM significantly decreases the IC_50_ values of mitoxantrone, SN-38 or topotecan in ABCG2-overexpressing cells. Previous studies have shown that mutations at position 482 in ABCG2 affects the inhibitory effect of certain ABCG2 modulators such as AC220 and novobiocin (Shiozawa et al., 2004; Li et al., 2017). AZ-628, according to our findings, showed the same level of antagonistic effect in both wild-type (R482) and mutants (R482G and R481T) ABCG2. Previous studies have reported several ABCG2 inhibitors with selective reversal effects on different ABCG2 mutants (Shiozawa et al., 2004; Li et al., 2017; Wang et al., 2020d). In our study, AZ-628 equally reversed MDR mediated by wild-type ABCG2 or mutated ABCG2. Additionally, AZ-628 does not affect MDR mediated by ABCB1, ABCC1, or ABCC10, which indicates the selective inhibitory effect of AZ-628. It is worth noting that, the cytotoxicity of AZ-628 was not significantly varied in parental and MDR cells, indicating that AZ-628, as an anticancer drug itself, was not affected by the MDR mediated by ABC transporters ABCB1, ABCG2, ABCC1, or ABCC10. These results showed that AZ-628 did not exhibit ABCB1-, ABCG2-, ABCC1-, or ABCC10-substrate features.

Reversal of ABCG2-mediated MDR may result from various reasons including modification of ABCG2 mRNA transcription or protein expression (Liao et al., 2019), alteration of intracellular localization of ABCG2 (Yu et al., 2020), modulation of signaling pathways (Zhang et al., 2020a), interaction with ABCG2-associated ATPase (Wang et al., 2020a) and direct interaction with the drug binding sites of ABCG2 (Cai et al., 2020; Wang et al., 2020a). In this study, we tested the effect of AZ-628 on the protein expression level as well as intracellular localization of ABCG2. The results showed that AZ-628 does not affect the protein expression after incubation for 72 h with a maximum of AZ-628 concentration of 5 μM. Moreover, the immunofluorescence results indicated that AZ-628 does not affect the intracellular localization of ABCG2. We suggested that the reversal effect of AZ-628 was not due to alteration of ABCG2 protein level or distribution.

The function of ABC transporters relies on ATP hydrolysis by ATPase (Ambudkar, 1998; Dean, 2001; Gottesman et al., 2002). Previous studies have shown that ABCG2 substrate drugs, such as topotecan, methotrexate or prazosin, stimulate ATPase activity (Glavinas et al., 2007). These drugs bind to the high affinity binding sites of transmembrane regions of ABCG2 (Gallus et al., 2014). In our results, AZ-628 stimulated ABCG2-associated ATPase to a maximum of 4.3-fold, indicating that AZ-628 also interacts with the substrate-drug binding sites and may compete with substrate drugs thus affecting their efflux. The stimulatory effects of AZ-628 to ABCG2 ATPase was comparable to a recently discovered ABCG2 inhibitor M3814 (Wu et al., 2020b), which further supported the interaction between AZ-628 and ABCG2 ATPase. The docking and MD simulation also showed that AZ-628 bonds to the same site as ABCG2 substrates with high score and stability, which consistently supported the conclusion. It is worth noting that although our results indicated that AZ-628 showed a substrate-like behavior, our MTT results showed AZ-628 was not a substrate of ABCG2. Actually, other ABC transporter inhibitors, like tariquidar (an ABCB1 inhibitor), showed similar behavior. Tariquidar stimulates ABCB1-associated ATPase activity and interacts with the drug binding sites of ABCB1, but was not a substrate of ABCB1. One possible explanation is that tariquidar traps the transporter at a specific conformation and thus blocks the transport function without being pumped out (Weidner et al., 2016). Thus, it is possible that AZ-628 is an ABCG2-inhibitor but not an ABCG2-substrate.

A recent study revealed that overexpression of ABC transporters was responsible for acquired resistance to antibody-drug conjugate (ADC) in cancer immunotherapy, and combinational administration of ABC transporter inhibitors increased the response rate of immunotherapy in resistant cancer cells (Chen et al., 2020). Moreover, such combinational therapy has been subjected to a phase I clinical trial (Chen et al., 2020). Therefore, finding novel ABC transporter inhibitors is critical for preclinical and clinical cancer research.

Collectively, this study provided evidence that AZ-628, at non-toxic concentrations, can sensitize ABCG2-mediated MDR cells by inhibiting the efflux function of ABCG2 and increase intracellular accumulation of ABCG2 substrate anticancer drugs. It would provide a rational in clinical applications using combination of AZ-628 for overcoming ABCG2-mediated MDR cancers.

## Data Availability Statement

The original contributions presented in the study are included in the article/supplementary material, further inquiries can be directed to the corresponding author/s.

## Author Contributions

Z-SC, D-HY, Y-FF, and J-QW designed the experiments. J-QW wrote the original draft manuscript and analyzed the data. Z-SC and D-HY reviewed the manuscript. J-QW, NJ, QC, HF, LL, Z-NL, and Q-XT performed the experiments. Z-SC and Y-FF edited the manuscript. All authors discussed the data and approved the final manuscript.

## Conflict of Interest

The authors declare that the research was conducted in the absence of any commercial or financial relationships that could be construed as a potential conflict of interest.

## References

[B1] Ambudkar S. V. (1998). Drug-stimulatable ATPase activity in crude membranes of human MDR1-transfected mammalian cells. *Methods Enzymol.* 292 504–514. 10.1016/S0076-6879(98)92039-0 9711578

[B2] Borst P. Elferink R. O. (2002). Mammalian ABC transporters in health and disease. *Annu. Rev. Biochem.* 71 537–592. 10.1146/annurev.biochem.71.102301.093055 12045106

[B3] Cai C.-Y. Zhai H. Lei Z.-N. Tan C.-P. Chen B.-L. Du Z.-Y. (2019). Benzoyl indoles with metabolic stability as reversal agents for ABCG2-mediated multidrug resistance. *Eur. J. Med. Chem.* 179 849–862. 10.1016/j.ejmech.2019.06.066 31302589 PMC6718313

[B4] Cai C.-Y. Zhang W. Wang J.-Q. Lei Z.-N. Zhang Y.-K. Wang Y.-J. (2020). Biological evaluation of non-basic chalcone CYB-2 as a dual ABCG2/ABCB1 inhibitor. *Biochem. Pharmacol.* 175:113848. 10.1016/j.bcp.2020.113848 32044354

[B5] Chen R. Herrera A. F. Hou J. Chen L. Wu J. Guo Y. (2020). Inhibition of MDR1 overcomes resistance to brentuximab vedotin in hodgkin lymphoma. *Clin. Cancer Res.* 26 1034–1044. 10.1158/1078-0432.CCR-19-1768 31811017 PMC7056527

[B6] Cui Q. Cai C.-Y. Wang J.-Q. Zhang S. Gupta P. Ji N. (2019a). Chk1 Inhibitor MK-8776 restores the sensitivity of chemotherapeutics in P-glycoprotein overexpressing cancer cells. *Int. J. Mol. Sci.* 20:4095. 10.3390/ijms20174095 31443367 PMC6747525

[B7] Cui Q. Yang Y. Ji N. Wang J.-Q. Ren L. Yang D.-H. (2019b). Gaseous signaling molecules and their application in resistant cancer treatment: from invisible to visible. *Future Med. Chem.* 11 323–336. 10.4155/fmc-2018-0403 30802141

[B8] Cui Q. Wang J.-Q. Assaraf Y. G. Ren L. Gupta P. Wei L. (2018). Modulating ROS to overcome multidrug resistance in cancer. *Drug Resist. Updat.* 41 1–25. 10.1016/j.drup.2018.11.001 30471641

[B9] Dean M. (2001). The human ATP-binding cassette (ABC) transporter superfamily. *Genome Res.* 11 1156–1166. 10.1101/gr.GR-1649R 11435397

[B10] Dlugosz A. Janecka A. (2016). ABC transporters in the development of multidrug resistance in cancer therapy. *Curr. Pharm. Des.* 22 4705–4716. 10.2174/1381612822666160302103646 26932159

[B11] Dong X.-D. Zhang M. Ma X. Wang J.-Q. Lei Z.-N. Teng Q.-X. (2020). Bruton’s tyrosine kinase (BTK) inhibitor RN486 overcomes ABCB1-mediated multidrug resistance in cancer cells. *Front. Cell Dev. Biol.* 8:865. 10.3389/fcell.2020.00865 32984343 PMC7481333

[B12] Feng W. Zhang M. Wu Z.-X. Wang J.-Q. Dong X.-D. Yang Y. (2020). Erdafitinib antagonizes ABCB1-mediated multidrug resistance in cancer cells. *Front. Oncol.* 10:955. 10.3389/fonc.2020.00955 32670878 PMC7330633

[B13] Friedrich R. E. Punke C. Reymann A. (2004). Expression of multi-drug resistance genes (mdr1, mrp1, bcrp) in primary oral squamous cell carcinoma. *In Vivo* 18 133–147. 15113040

[B14] Gallus J. Juvale K. Wiese M. (2014). Characterization of 3-methoxy flavones for their interaction with ABCG2 as suggested by ATPase activity. *Biochim. Biophys. Acta* 1838 2929–2938. 10.1016/j.bbamem.2014.08.003 25128152

[B15] Glavinas H. Kis E. Pál A. Kovács R. Jani M. Vági E. (2007). ABCG2 (breast cancer resistance protein/mitoxantrone resistance-associated protein) ATPase assay: a useful tool to detect drug-transporter interactions. *Drug Metab. Dispos.* 35 1533–1542. 10.1124/dmd.106.014605 17537873

[B16] Gottesman M. M. Ambudkar S. V. (2001). Overview: ABC transporters and human disease. *J. Bioenerg. Biomembr.* 33 453–458. 11804186 10.1023/a:1012866803188

[B17] Gottesman M. M. Fojo T. Bates S. E. (2002). Multidrug resistance in cancer: role of ATP–dependent transporters. *Nat. Rev. Cancer* 2 48–58. 10.1038/nrc706 11902585

[B18] Halilovic E. Solit D. B. (2008). Therapeutic strategies for inhibiting oncogenic BRAF signaling. *Curr. Opin. Pharmacol.* 8 419–426. 10.1016/j.coph.2008.06.014 18644254

[B19] Honjo Y. Hrycyna C. A. Yan Q. W. Medina-Pérez W. Y. Robey R. W. van de Laar A. (2001). Acquired mutations in the MXR/BCRP/ABCP gene alter substrate specificity in MXR/BCRP/ABCP-overexpressing cells. *Cancer Res.* 61 6635–6639. 11559526

[B20] Jackson S. M. Manolaridis I. Kowal J. Zechner M. Taylor N. M. I. Bause M. (2018). Structural basis of small-molecule inhibition of human multidrug transporter ABCG2. *Nat. Struct. Mol. Biol.* 25 333–340. 10.1038/s41594-018-0049-1 29610494

[B21] Ji N. Yang Y. Cai C.-Y. Lei Z.-N. Wang J.-Q. Gupta P. (2019a). Selonsertib (GS-4997), an ASK1 inhibitor, antagonizes multidrug resistance in ABCB1- and ABCG2-overexpressing cancer cells. *Cancer Lett.* 440–441 82–93. 10.1016/j.canlet.2018.10.007 30315846 PMC8132112

[B22] Ji N. Yang Y. Cai C.-Y. Wang J.-Q. Lei Z.-N. Wu Z.-X. (2019b). Midostaurin reverses ABCB1-mediated multidrug resistance, an in vitro study. *Front. Oncol.* 9:514. 10.3389/fonc.2019.00514 31275850 PMC6591272

[B23] Ji N. Yang Y. Lei Z.-N. Cai C.-Y. Wang J.-Q. Gupta P. (2018). Ulixertinib (BVD-523) antagonizes ABCB1- and ABCG2-mediated chemotherapeutic drug resistance. *Biochem. Pharmacol.* 158 274–285. 10.1016/j.bcp.2018.10.028 30431011

[B24] Kovalev A. A. Tsvetaeva D. A. Grudinskaja T. V. (2013). Role of ABC-cassette transporters (MDR1, MRP1, BCRP) in the development of primary and acquired multiple drug resistance in patients with early and metastatic breast cancer. *Exp. Oncol.* 35 287–290. 24382439

[B25] Lage H. (2016). Gene therapeutic approaches to overcome ABCB1-mediated drug resistance. *Recent Results Cancer Res.* 209 87–94. 10.1007/978-3-319-42934-2_6 28101689

[B26] Li J. Kumar P. Anreddy N. Zhang Y.-K. Wang Y.-J. Chen Y. (2017). Quizartinib (AC220) reverses ABCG2-mediated multidrug resistance: In vitro and in vivo studies. *Oncotarget* 8 93785–93799. 10.18632/oncotarget.21078 29212189 PMC5706835

[B27] Liao D. Zhang W. Gupta P. Lei Z.-N. Wang J.-Q. Cai C.-Y. (2019). Tetrandrine interaction with ABCB1 reverses multidrug resistance in cancer cells through competition with anti-cancer drugs followed by downregulation of ABCB1 expression. *Molecules* 24:4383. 10.3390/molecules24234383 31801248 PMC6930469

[B28] Luo X. Teng Q.-X. Dong J.-Y. Yang D.-H. Wang M. Dessie W. (2020). Antimicrobial peptide reverses ABCB1-mediated chemotherapeutic drug resistance. *Front. Pharmacol.* 11:1208. 10.3389/fphar.2020.01208 32903706 PMC7438908

[B29] Mao Q. Unadkat J. D. (2015). Role of the breast cancer resistance protein (BCRP/ABCG2) in drug transport–an update. *AAPS J.* 17 65–82. 10.1208/s12248-014-9668-6 25236865 PMC4287283

[B30] McDermott U. Sharma S. V. Dowell L. Greninger P. Montagut C. Lamb J. (2007). Identification of genotype-correlated sensitivity to selective kinase inhibitors by using high-throughput tumor cell line profiling. *Proc. Natl. Acad. Sci. U.S.A.* 104 19936–19941. 10.1073/pnas.0707498104 18077425 PMC2148401

[B31] Morrow C. S. (2006). Multidrug resistance protein 1 (MRP1, ABCC1) mediates resistance to mitoxantrone via glutathione-dependent drug efflux. *Mol. Pharmacol.* 69 1499–1505. 10.1124/mol.105.017988 16434618

[B32] Narayanan S. Gujarati N. A. Teng Q. Wang J. Cai C.-Y. Yang Y. (2020). “Abstract 3010: VKNG 1 reverses multidrug resistance by inhibiting ABCG2 mediated drug transport in vitro and in vivo,” in *Proceedings of the AACR Annual Meeting 2020*, Philadelphia, PA, 10.1158/1538-7445.AM2020-3010

[B33] Polgar O. Robey R. W. Bates S. E. (2008). ABCG2: structure, function and role in drug response. *Expert. Opin. Drug. Metab. Toxicol.* 4 1–15. 10.1517/17425255.4.1.1 18370855

[B34] Robey R. W. Medina-Pérez W. Y. Nishiyama K. Lahusen T. Miyake K. Litman T. (2001). Overexpression of the ATP-binding cassette half-transporter, ABCG2 (Mxr/BCrp/ABCP1), in flavopiridol-resistant human breast cancer cells. *Clin. Cancer Res.* 7 145–152. 11205902

[B35] Ross D. D. Karp J. E. Chen T. T. Doyle L. A. (2000). Expression of breast cancer resistance protein in blast cells from patients with acute leukemia. *Blood* 96 365–368. 10.1182/blood.V96.1.365 10891476

[B36] Shiozawa K. Oka M. Soda H. Yoshikawa M. Ikegami Y. Tsurutani J. (2004). Reversal of breast cancer resistance protein (BCRP/ABCG2)-mediated drug resistance by novobiocin, a coumermycin antibiotic. *Int. J. Cancer* 108 146–151. 10.1002/ijc.11528 14618629

[B37] Taylor N. M. I. Manolaridis I. Jackson S. M. Kowal J. Stahlberg H. Locher K. P. (2017). Structure of the human multidrug transporter ABCG2. *Nature* 546 504–509. 10.1038/nature22345 28554189

[B38] Teng Q.-X. Luo X. Lei Z.-N. Wang J.-Q. Wurpel J. Qin Z. (2020). The multidrug resistance-reversing activity of a novel antimicrobial peptide. *Cancers* 12:1963. 10.3390/cancers12071963 32707710 PMC7409168

[B39] Teodori E. Dei S. Martelli C. Scapecchi S. Gualtieri F. (2006). The functions and structure of ABC transporters: implications for the design of new inhibitors of Pgp and MRP1 to control multidrug resistance (MDR). *Curr. Drug Targets* 7 893–909. 10.2174/138945006777709520 16842220

[B40] Trott O. Olson A. J. (2009). AutoDock Vina: improving the speed and accuracy of docking with a new scoring function, efficient optimization, and multithreading. *J. Comput. Chem.* 31 455–461. 10.1002/jcc.21334 19499576 PMC3041641

[B41] Ullah M. F. (2008). Cancer multidrug resistance (MDR): a major impediment to effective chemotherapy. *Asian Pac. J. Cancer Prev.* 9 1–6. 18439063

[B42] Wang J. Wang J.-Q. Cai C.-Y. Cui Q. Yang Y. Wu Z.-X. (2020a). Reversal effect of ALK inhibitor NVP-TAE684 on ABCG2-overexpressing cancer cells. *Front. Oncol.* 10:228. 10.3389/fonc.2020.00228 32175279 PMC7056829

[B43] Wang J. Yang D.-H. Yang Y. Wang J.-Q. Cai C.-Y. Lei Z.-N. (2020b). Overexpression of ABCB1 transporter confers resistance to mTOR inhibitor WYE-354 in cancer cells. *Int. J. Mol. Sci.* 21 1387. 10.3390/ijms21041387 32092870 PMC7073023

[B44] Wang J.-Q. Lei Z.-N. Teng Q.-X. Wang B. Ma L.-Y. Liu H.-M. (2020c). “Abstract 2983: a synthetic derivative of 1,2,3-triazole-pyrimidine hybrid reverses multidrug resistance mediated by MRP7,” in *Proceedings of the AACR Annual Meeting 2020*, Philadelphia, PA, 10.1158/1538-7445.AM2020-2983

[B45] Wang J.-Q. Li J. Y. Teng Q.-X. Lei Z.-N. Ji N. Cui Q. (2020d). Venetoclax, a BCL-2 inhibitor, enhances the efficacy of chemotherapeutic agents in wild-type ABCG2-overexpression-mediated MDR cancer cells. *Cancers* 12:466. 10.3390/cancers12020466 32085398 PMC7072352

[B46] Wang J.-Q. Wang B. Lei Z.-N. Teng Q.-X. Li J. Y. Zhang W. (2019). Derivative of 5-cyano-6-phenylpyrimidin antagonizes ABCB1- and ABCG2-mediated multidrug resistance. *Eur. J. Pharmacol.* 863:172611. 10.1016/j.ejphar.2019.172611 31476282

[B47] Ween M. P. Armstrong M. A. Oehler M. K. Ricciardelli C. (2015). The role of ABC transporters in ovarian cancer progression and chemoresistance. *Crit. Rev. Oncol. Hematol.* 96 220–256. 10.1016/j.critrevonc.2015.05.012 26100653

[B48] Weidner L. D. Fung K. L. Kannan P. Moen J. K. Kumar J. S. Mulder J. (2016). Tariquidar is an inhibitor and not a substrate of human and mouse P-glycoprotein. *Drug. Metab. Dispos.* 44 275–282. 10.1124/dmd.115.067785 26658428 PMC4746486

[B49] Wu Z. Yang Y. Wang G. Wang J. Teng Q. Sun L. (2020a). Dual TTK/CLK2 inhibitor, CC-671, selectively antagonizes ABCG2-mediated multidrug resistance in lung cancer cells. *Cancer Sci.* 111 2872–2882. 10.1111/cas.14505 32478948 PMC7419038

[B50] Wu Z.-X. Peng Z. Yang Y. Wang J.-Q. Teng Q.-X. Lei Z.-N. (2020b). M3814, a DNA-PK inhibitor, modulates ABCG2-mediated multidrug resistance in lung cancer cells. *Front. Oncol.* 10:674. 10.3389/fonc.2020.00674 32477940 PMC7235170

[B51] Wu Z.-X. Yang Y. Teng Q.-X. Wang J.-Q. Lei Z.-N. Wang J.-Q. (2020c). Tivantinib, A c-Met inhibitor in clinical trials, is susceptible to ABCG2-mediated drug resistance. *Cancers* 12:186. 10.3390/cancers12010186 31940916 PMC7017082

[B52] Wu Z.-X. Teng Q.-X. Cai C.-Y. Wang J.-Q. Lei Z.-N. Yang Y. (2019). Tepotinib reverses ABCB1-mediated multidrug resistance in cancer cells. *Biochem. Pharmacol.* 166 120–127. 10.1016/j.bcp.2019.05.015 31078601

[B53] Yang Y. Ji N. Cai C. Wang J. Lei Z. Teng Q. (2020a). Modulating the function of ABCB1: in vitro and in vivo characterization of sitravatinib, a tyrosine kinase inhibitor. *Cancer Commun.* 40 285–300. 10.1002/cac2.12040 32525624 PMC7365458

[B54] Yang Y. Ji N. Teng Q.-X. Cai C.-Y. Wang J.-Q. Wu Z.-X. (2020b). Sitravatinib, a tyrosine kinase inhibitor, inhibits the transport function of ABCG2 and restores sensitivity to chemotherapy-resistant cancer cells in vitro. *Front. Oncol.* 10:700 10.3389/fonc.2020.00700 PMC7236772 32477943

[B55] Yoh K. Ishii G. Yokose T. Minegishi Y. Tsuta K. Goto K. (2004). Breast cancer resistance protein impacts clinical outcome in platinum-based chemotherapy for advanced non-small cell lung cancer. *Clin. Cancer Res.* 10 1691–1697. 10.1158/1078-0432.ccr-0937-3 15014021

[B56] Yu T. Cheng H. Ding Z. Wang Z. Zhou L. Zhao P. (2020). GPER mediates decreased chemosensitivity via regulation of ABCG2 expression and localization in tamoxifen-resistant breast cancer cells. *Mol. Cell. Endocrinol.* 506:110762. 10.1016/j.mce.2020.110762 32087276

[B57] Zaher H. Khan A. A. Palandra J. Brayman T. G. Yu L. Ware J. A. (2006). Breast cancer resistance protein (Bcrp/abcg2) is a major determinant of sulfasalazine absorption and elimination in the mouse. *Mol. Pharm.* 3 55–61. 10.1021/mp050113v 16686369

[B58] Zhang L. Li Y. Wang Q. Chen Z. Li X. Wu Z. (2020a). The PI3K subunits, P110α and P110β are potential targets for overcoming P-gp and BCRP-mediated MDR in cancer. *Mol. Cancer* 19:10. 10.1186/s12943-019-1112-1 31952518 PMC6966863

[B59] Zhang M. Chen X.-Y. Dong X.-D. Wang J.-Q. Feng W. Teng Q.-X. (2020b). NVP-CGM097, an HDM2 inhibitor, antagonizes ATP-binding cassette subfamily B member 1-mediated drug resistance. *Front. Oncol.* 10:1219 10.3389/fonc.2020.01219 PMC7390918 32793491

[B60] Zhang W. Fan Y.-F. Cai C.-Y. Wang J.-Q. Teng Q.-X. Lei Z.-N. (2018). Olmutinib (BI1482694/HM61713), a novel epidermal growth factor receptor tyrosine kinase inhibitor, reverses ABCG2-mediated multidrug resistance in cancer cells. *Front. Pharmacol.* 9:1097. 10.3389/fphar.2018.01097 30356705 PMC6189370

[B61] Zhang Y.-K. Wang Y.-J. Gupta P. Chen Z.-S. (2015). Multidrug resistance proteins (MRPs) and cancer therapy. *AAPS J.* 17 802–812. 10.1208/s12248-015-9757-1 25840885 PMC4476997

